# Rod pathway and cone pathway retinal dysfunction in the 5xFAD mouse model of Alzheimer’s disease

**DOI:** 10.1038/s41598-021-84318-2

**Published:** 2021-03-01

**Authors:** J. Jason McAnany, Nathanael Matei, Yi-Fan Chen, Karen Liu, Jason C. Park, Mahnaz Shahidi

**Affiliations:** 1 Department of Ophthalmology and Visual Sciences, University of Illinois at Chicago, Chicago, IL USA; 2 Department of Ophthalmology, University of Southern California, Los Angeles, CA USA; 3 Center for Clinical and Translational Science, University of Illinois at Chicago, Chicago, IL USA

**Keywords:** Biomarkers, Alzheimer's disease, Eye diseases

## Abstract

To characterize rod- and cone-pathway function in the 5xFAD mouse model of Alzheimer’s disease (AD) using the full-field electroretinogram (ERG). Dark-adapted (DA; rod-pathway) and light-adapted (LA; cone-pathway) ERGs were recorded from three-month-old 5xFAD and wild type (WT) mice. ERGs were elicited by achromatic flashes (0.01–25 cd-s-m^−^^2^). Amplitude and implicit time (IT) of the a-wave, b-wave, and oscillatory potentials (OPs) were calculated according to convention. In addition, the amplitude and IT of the photopic negative response (PhNR) were measured from the LA recordings. Amplitude and IT differences between the 5xFAD and WT groups were evaluated using quantile regression models. Under DA conditions, there were significant differences between the 5xFAD and WT groups in post-receptor function, whereas photoreceptor function did not differ significantly. Specifically, the DA a-wave amplitude did not differ between groups (*p* = 0.87), whereas the b-wave amplitude was reduced in the 5xFAD mice (*p* = 0.003). There were significant OP (*p* < 0.001) and a-wave (*p* = 0.04) delays, but the a-wave delay may be attributable to a post-receptor abnormality. Under LA conditions, the only 5xFAD abnormalities were in the PhNR, which was reduced (*p* = 0.009) and delayed (*p* = 0.04). The full-field ERG can be abnormal in the 5xFAD model of AD, with the greatest effects on post-receptor rod pathway function. These results indicate that retinal electrophysiology may be a useful tool for evaluating neural dysfunction in AD.

## Introduction

Alzheimer's disease (AD) is a progressive neurodegenerative disorder that is commonly associated with abnormal aggregation of amyloid beta (Aβ) and tau proteins in the brain. The primary focus of AD research has been on cortical pathophysiology and the corresponding deficits in memory and cognition, but there is accumulating evidence for retinal abnormalities as well (see Alber et al.^1^ for a recent review). Pathological effects of AD on the retina may be expected, as the retina and brain share important similarities, including embryologic origin and direct transfer of amyloid precursor protein (APP). Indeed, clinical studies have provided substantial evidence for abnormalities in both retinal structure^2–6^ and function^3,5–10^ in AD patients.

A number of animal models of AD have been developed that permit mechanistic studies and evaluating therapeutic compounds that are difficult to perform in human subjects^11–14^. Of the mouse models of AD available, the 5xFAD transgenic mouse is one of the more commonly used Aβ models^14^. In this mouse model, five distinct mutations within the amyloid precursor protein (APP) and presenilin 1 (PSEN1) genes are incorporated into a single transgenic line: APP KM670/671NL, APP I716V, APP V717I, PSEN1 M146L (A > C), PSEN1 L286V^15^. These mutations produce rapid amyloid pathology in the brain as early as 2 months of age^15^. In addition to cortical pathology, 5xFAD mice develop behavioral deficits that begin to manifest at approximately 4–5 months of age^15,16^.

Although cortical structure and function in the 5xFAD mouse has been studied extensively, there are relatively few reports of retinal structure and function in these animals. The available reports show Aβ peptide elevation in the retina^17–20^, consistent with the findings in the brain, which may occur as early as one month of age^18^. The electroretinogram (ERG) has been used to evaluate retinal function in this model^18,20–22^, with the most complete report of ERG findings provided by Lim et al.^22^ For 17-month-old 5xFAD mice, photoreceptor function (inferred from a-wave analyses), bipolar cell function (inferred from b-wave analyses), and ganglion cell function (inferred from the scotopic threshold response) were all significantly abnormal^22^. For younger mice (6–12 month-old), trends for bipolar cell abnormalities were apparent for stimulus luminance greater than approximately 0.001 cd-s-m^−2^, but these did not reach statistical significance. In contrast, ganglion cell function was significantly abnormal at all ages, as inferred from measurements of the scotopic threshold response. Similarly, Do et al.^21^ reported a statistically significant reduction in bipolar cell function in 6-month-old 5xFAD mice for stimuli greater than 0.001 cd-s-m^−2^. Do et al.^21^ did not describe photoreceptor or ganglion cell measures of function. Criscuolo et al.^18^ reported a trend for reduced bipolar cell function in 5xFAD mice at 1 to 6 months of age (stimuli of 0.1 and 20 cd-s-m^−2^), but these differences did not achieve statistical significance and no measure of photoreceptor function was provided. However, Criscuolo et al.^18^ did report reduced pattern ERG amplitude, a measure of retinal ganglion cell function, at all ages. Taken together, previous ERG studies in the 5xFAD mouse suggest: (1) photoreceptor function is normal at young ages, but reduced in older animals; (2) measures of retinal ganglion cell function are reduced at young ages (e.g. 1 month) and continue to decline with advancing age; (3) bipolar cell function can be reduced, but the extent of abnormality depends on stimulus luminance and may not become statistically significant until later ages.

Although previous work has informed our understanding of rod-pathway-mediated retinal dysfunction in the 5xFAD model of AD^18,21,22^, the extent to which the cone-pathway is affected is less clear. To date, the full-field ERG has not been assessed under light-adapted conditions in 5xFAD mice. It is possible that rod and cone pathway function are differentially affected, as reported in the APP_swe_/PS1ΔE9 mouse model of AD^23^. The purpose of the present study was to expand upon previous studies to characterize and compare retinal function under both light- and dark-adapted conditions in young (three-month-old) 5xFAD mice to provide insight into early rod- and cone-pathway dysfunction in this model. ERG measurements were performed across a broad range of stimulus luminance to gain a better understanding of how the extent of abnormality varies for different stimuli within the rod and cone pathways. In addition, a preliminary analysis was performed in a subset of animals to determine the relationship between Aβ concentration and ERG amplitude.

## Methods

### Animals

All procedures were approved by the University of Southern California Institutional Animal Care and Use Committee and adhered to both the ARRIVE guidelines and the articles of the statement of Use of Animals in Ophthalmic and Vision Research by the Association for Research in Vision and Ophthalmology. Twenty-two 5xFAD mice and 13 wild type (WT) mice were tested at three months of age. All mice were male and had black pigmentation (C57BL/6 background). The 5xFAD mouse strain was obtained from the Mutant Mouse Resource and Research Center at The Jackson Laboratory (B6.Cg-Tg (APPSwFlLon,PSEN1*M146L*L286V) 6799Vas/Mmjax, RRID:MMRRC_034848-JAX). Of note, it was confirmed that this strain does not carry the *Pde6b*^*rd1*^ retinal degeneration allele. The WT mice were obtained from The Jackson Laboratory (C57BL/6 J; N = 9) or Charles River Laboratories (C57BL/6; N = 4).

### ERG apparatus, stimuli, and procedure

All stimuli were generated and delivered using an Ocuscience electrophysiology system (HMsERG 2000; Henderson, Nevada). Stimuli consisted of LED-generated achromatic flashes that ranged from 0.01 to 25.0 cd-s-m^−2^ in 8 steps that were spaced approximately 0.5 log units apart. Flashes were presented in the dark after two hours of dark-adaptation or against a 30 cd/m^2^ achromatic background after 10 min of light-adaptation. Mice were anesthetized with ketamine (100 mg/kg) and xylazine (5 mg/kg). The pupil of the test eye was dilated with 1% tropicamide and 2.5% phenylephrine drops. A gold-embedded cornea electrode (Mayo Corporation, Japan) was used as the active electrode, which was referenced to a needle electrode placed in the cheek; a second needle electrode placed in the tail served as the ground.

### ERG analysis

Amplitude and implicit time were calculated according to convention^24,25^. Specifically, the a-wave was measured from the pre-flash baseline (0 µV) to the trough of the response. This measure largely represents photoreceptor function, with additional contributions from bipolar cells. A secondary analysis measured the a-wave amplitude at a fixed time following the flash (6 ms under dark-adapted conditions and 5 ms under light-adapted conditions). Measuring the a-wave at a fixed time shortly after the flash helps to minimize contributions from post-receptor neurons^24^. The b-wave was measured from the trough of the a-wave to the peak of the b-wave and represents bipolar cell activity. Oscillatory potentials (OPs) were extracted from the responses to each flash using a conventional finite impulse response band-pass filter (70–300 Hz passband) that is described in detail elsewhere^26^. Four prominent OPs were apparent for each flash luminance and the individual trough-to-peak amplitude for each OP was measured. These four amplitude values were averaged to provide a single mean OP (mOP) amplitude for each stimulus flash luminance. Likewise, the peak time of each OP was measured and summed to provide a single measure of OP timing at each stimulus flash luminance. The OPs are thought to arise primarily from interactions among inner-retinal neurons^27,28^, but their source has not been fully established. The photopic negative response (PhNR) was measured for responses obtained under light-adapted conditions. The PhNR is a slow negative potential that follows the b-wave and is thought to be generated primarily by retinal ganglion cells^29–31^, although previous studies in mice indicate that glia may contribute to the response^29^. The PhNR was measured from the pre-stimulus baseline (0 µV) to the trough of the PhNR^30^.

All statistical analyses were conducted using R (version 4.0.2; R Core Team, Vienna, Austria) and SigmaPlot (version 12; San Jose, CA, USA). Normality of the amplitude and implicit time distributions were evaluated using Shapiro–Wilk tests and quantile–quantile plots; both distributions were found to be skewed (both *p* > 0.05). Consequently, linear quantile mixed models (LQMMs)^32,33^, which are based on median values, were used to compare the 5xFAD and WT amplitude and IT data. In each model, group (5xFAD vs WT) was included as the main independent variable while adjusting for flash luminance. A random intercept was added at the animal level to account for the repeated measures obtained from each animal. For waveform components that returned a significant difference (*p* < 0.05) between the two groups, univariate linear quantile models (LQMs)^34,35^ were developed to determine whether the groups differed at the 8 different flash luminances. These analyses were stratified by response component (a-wave, b-wave, OP, and PhNR) and adaptation condition (light-adapted and dark-adapted). For the LQMs, Bonferroni correction was used and two-sided p-values less than 0.0063 (0.05/8) were considered to be statistically significant.

## Results

### Dark-adapted responses to evaluate rod-pathway function

**Figure 1:**
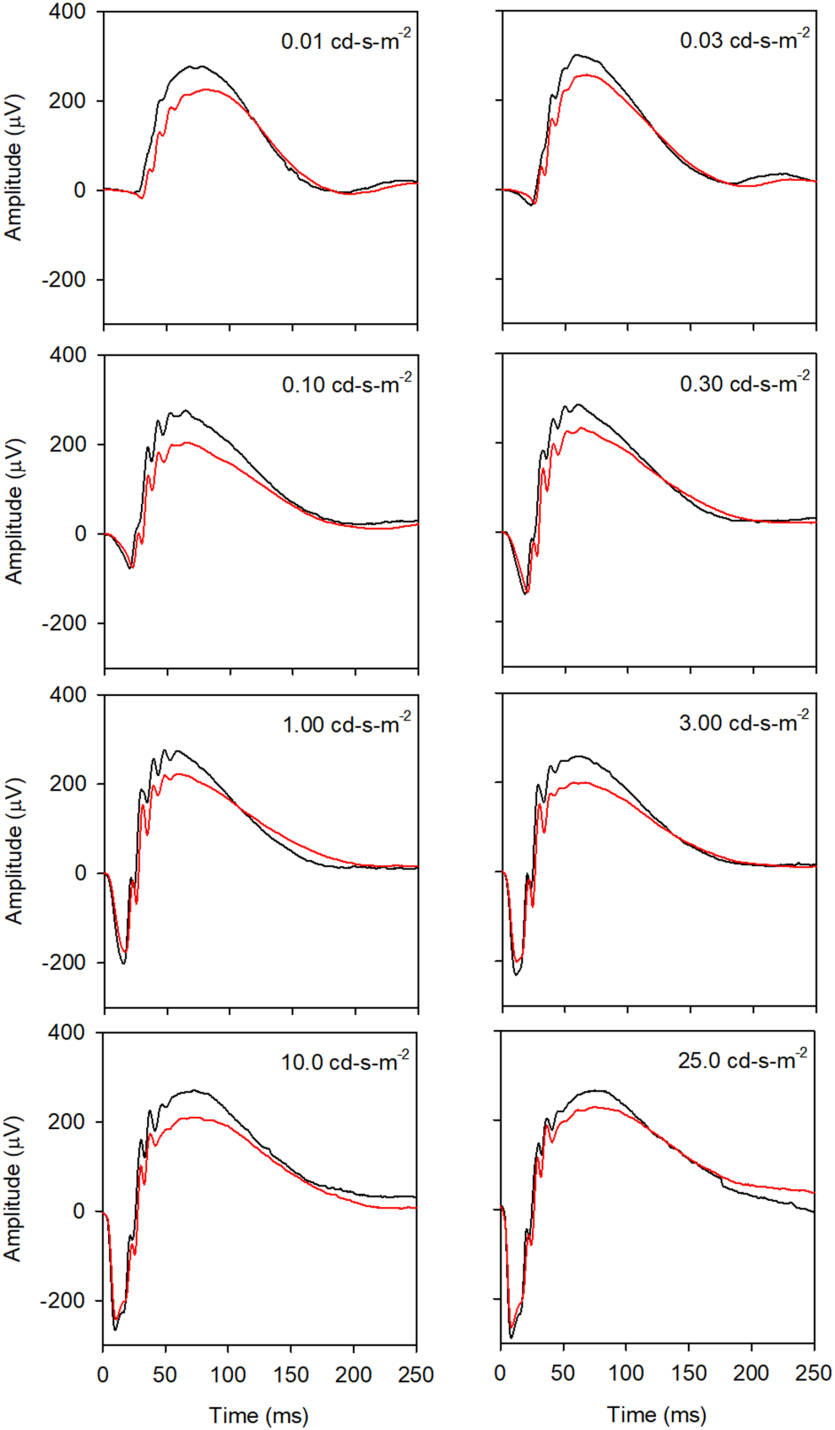
Mean waveforms for the WT (black) and 5xFAD (red) mice are shown for the 8 different flash luminances (indicated in each panel) under dark-adapted conditions.

**Figure 2:**
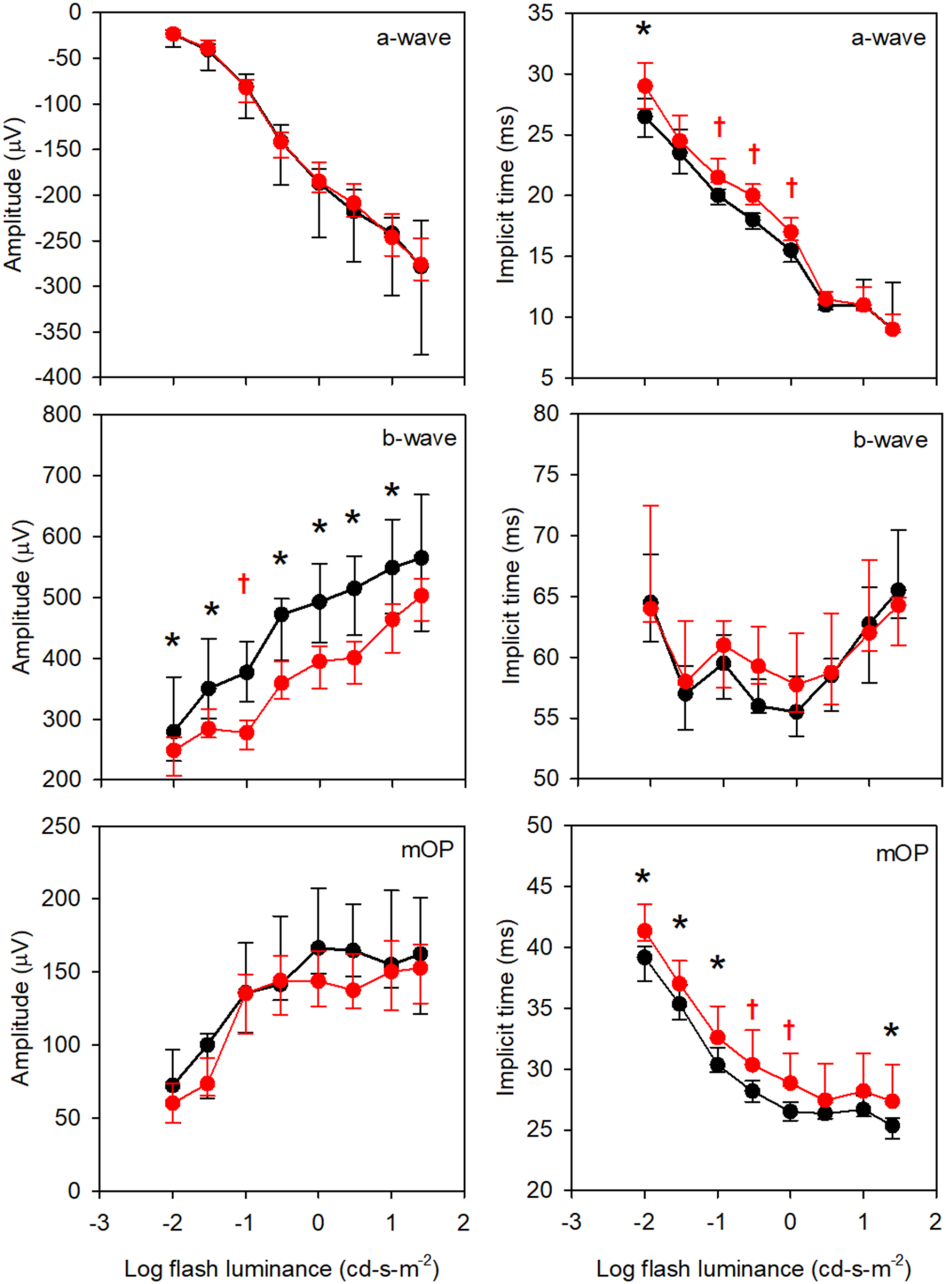
Median response amplitude (left column) and implicit time (right column) for the WT (black) and 5xFAD (red) mice as a function of log flash luminance. Measurements were obtained under dark-adapted conditions. Error bars represent 95% confidence intervals derived by bootstrapping. Each row represents a different waveform component, as indicated in the panels. The asterisks mark statistically significant differences between the WT and 5xFAD groups at the *p* = 0.05 level, whereas daggers represent Bonferroni corrected differences at the *p* = 0.0063 level (*p* = 0.05/8).

Figure 1 shows the mean ERG waveforms obtained under dark-adapted conditions for the WT (black) and 5xFAD (red) mice. Each panel shows the response elicited by a different flash luminance. The average waveforms show that the b-wave is consistently reduced for the 5xFAD mice relative to the WT mice, whereas the a-waves appear generally similar for the two groups. These waveforms provide an overview of the response shape for the two groups; individual waveform components were measured and quantified in Fig. 2.

**Figure 3:**
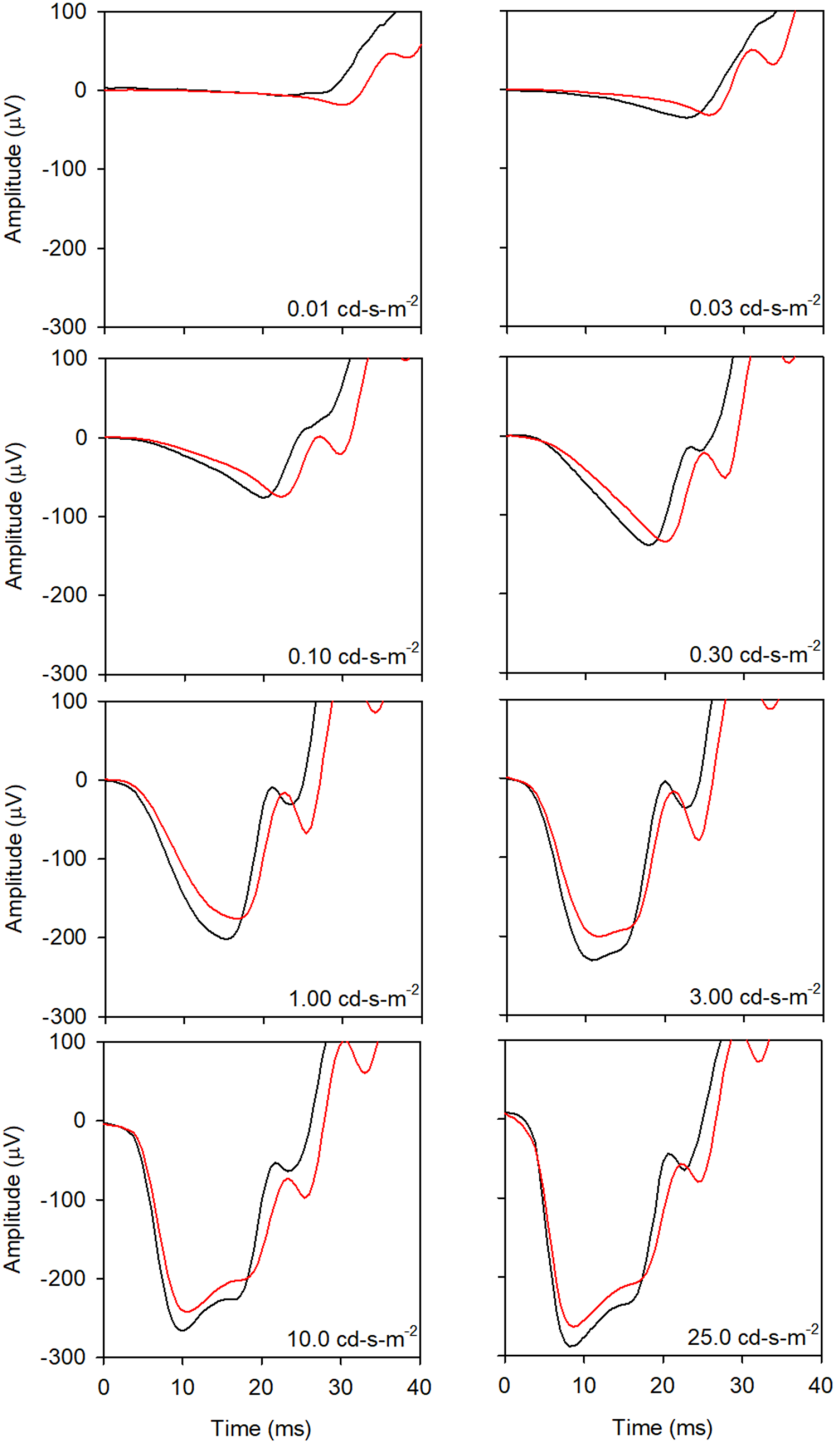
Mean waveforms at each flash luminance are replotted from Fig. 1 on expanded time and amplitude scales to highlight the a-waves. All other conventions are as in Fig. 1.

Figure 2 shows the median amplitudes (first column) and ITs (second column) for the WT (black) and 5xFAD (red) mice under dark-adapted conditions. Error bars represent 95% confidence intervals, derived by bootstrapping. The first row (left) shows that the median a-wave amplitudes for the WT and 5xFAD mice were highly similar. Indeed, the LQMM indicated no significant difference between the groups (median difference of 2.59 µV; *p* = 0.87). In contrast, the a-wave IT was slightly delayed for the 5xFAD group compared to the controls, primarily at low to moderate flash luminances. The LQMM indicated a small but statistically significant difference between the groups (median difference of 1.01 ms; *p* = 0.04). LQMs indicated that the WT and 5xFAD amplitudes differed for flash luminances of 0.01, and 0.10 to 1.0 cd-s-m^−2^ (all *p* < 0.05; indicated by the symbols). Following Bonferroni correction, the WT and 5xFAD amplitudes differed significantly for the 0.1 to 1.0 cd-s-m^−2^ flash luminances (*p* < 0.006; indicated by the daggers). Additional data concerning the small a-wave IT delays are presented in Fig. 3.

The second row of Fig. 2 (left) shows that the median b-wave amplitude for the 5xFAD mice was significantly smaller than that of the WT mice (median difference of 130.49 µV; *p* = 0.003). LQMs indicated that the WT and 5xFAD amplitudes differed for flash luminances of 0.01 to 10.0 cd-s-m^−2^ (all *p* < 0.05; indicated by the symbols). Following Bonferroni correction, the WT and 5xFAD amplitudes differed significantly only for the 0.1 cd-s-m^−2^ flash luminance (*p* < 0.0001; indicated by the dagger). There was no significant difference in b-wave IT between the groups (median difference of 0.91 ms; *p* = 0.51). Preliminary analyses performed in a subset of animals showed that the dark-adapted 3.0 cd-s-m^−2^ b-wave amplitude was correlated with the concentration of retinal Aβ42 (Spearman’s ρ = -0.68, *p* = 0.019; Supplementary Materials).

The third row of Fig. 2 (left) shows that the median OP amplitude for the 5xFAD mice was similar to that of the WT mice (median difference of 27.16 µV; *p* = 0.19). There was, however, a significant delay in OP IT between the groups (median difference of 3.17 ms; *p* < 0.001). LQMs indicated that the WT and 5xFAD ITs differed for flash luminances between 0.01 to 1.0 cd-s-m^−2^, and at 25.0 cd-s-m^−2^ (all *p* < 0.05; indicated by the symbols). Following Bonferroni correction, the WT and 5xFAD ITs differed significantly for flash luminances of 0.30 and 1.0 cd-s-m^−2^ (both *p* < 0.006; indicated by the daggers).

Figure 3 replots the waveforms of Fig. 1 at expanded time and voltage scales to more clearly visualize the dark-adapted a-waves. This figure shows that the 5xFAD a-wave trough was delayed for low flash luminances (0.01 to 0.30 cd-s-m^−2^), but the amplitude was highly similar to that of the WT mice. For higher flash luminances, the a-wave IT was generally normal for the 5xFAD mice, but the amplitude was slightly reduced. Given the likelihood for post-receptor contributions to the later time points of the a-wave^24,36,37^, the a-wave was re-measured at an earlier time point (6 ms following the flash), which helps to minimize these contributions. An LQMM indicated no significant difference between the groups in a-wave amplitude measured at 6 ms following the flash (median difference of 5.69 µV *p* = 0.15), which is consistent with the finding obtained for the a-wave amplitude measured at the trough.

### Light-adapted responses to evaluate cone-pathway function

**Figure 4:**
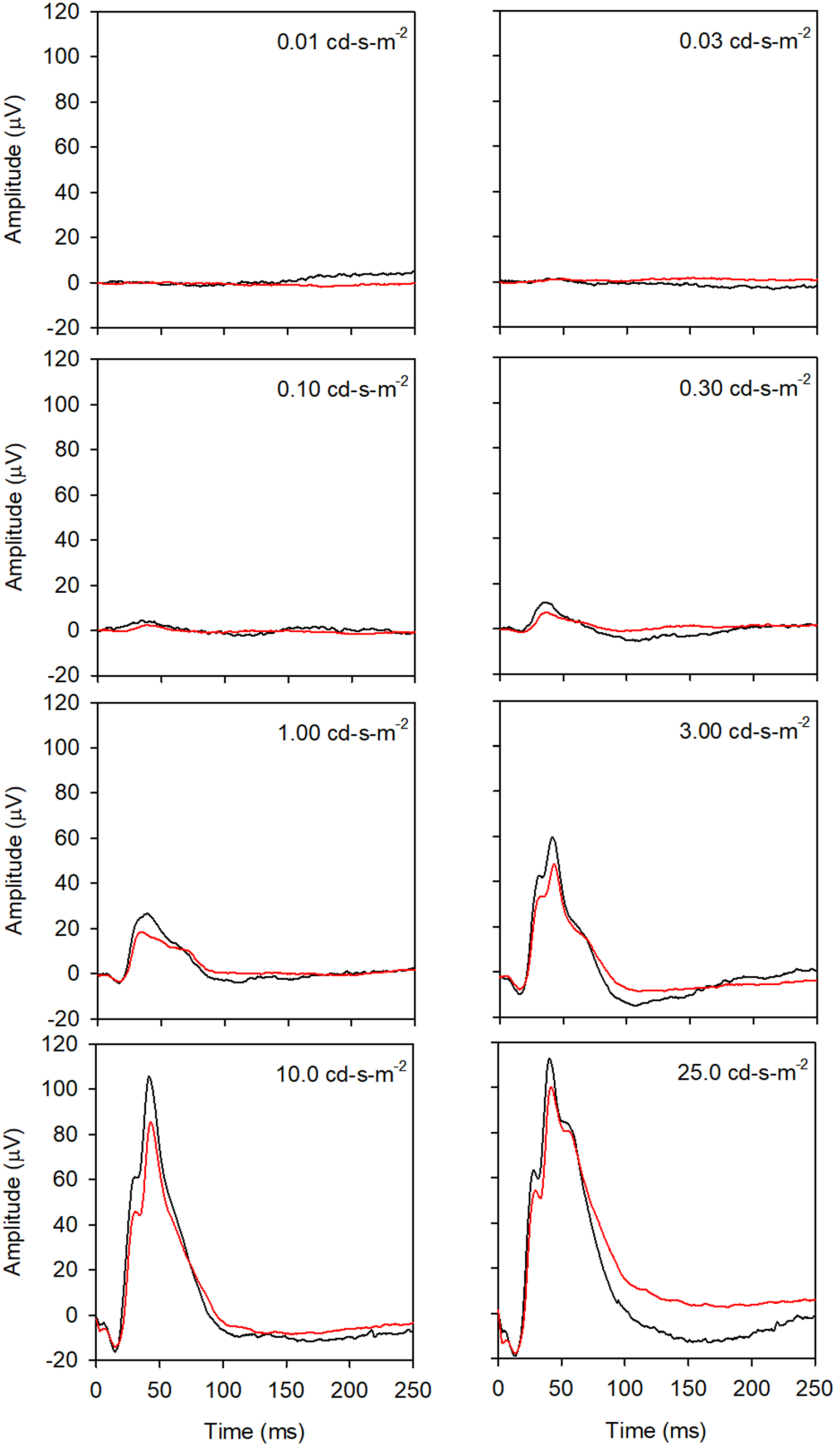
Mean waveforms for the WT (black) and 5xFAD (red) mice are shown for the 8 different flash luminances (indicated in each panel) under light-adapted conditions.

Figure 4 shows the mean ERG waveforms obtained under light-adapted conditions for the WT (black) and 5xFAD (red) mice. Each panel shows the response elicited by a different flash luminance. For flash luminance less than 0.10 cd-s-m^−2^, there was little or no measurable response. For higher flash luminances, responses became apparent, but the differences between the 5xFAD and WT mice were relatively small. For select flash luminances (e.g. 1.0, 3.0, and 10.0 cd-s-m^−2^), the mean b-wave was somewhat smaller for the 5xFAD group compared to the WT. The PhNR (the slow negative component following the b-wave), was clearly reduced for the 5xFAD group relative to the WT group for the 25 cd-s-m^−2^ flash. In fact, the PhNR was absent, or nearly absent, for the 5xFAD group for all flash luminances; however, the PhNR was also generally small for the WT mice for flash luminances below 25 cd-s-m^−2^.

**Figure 5:**
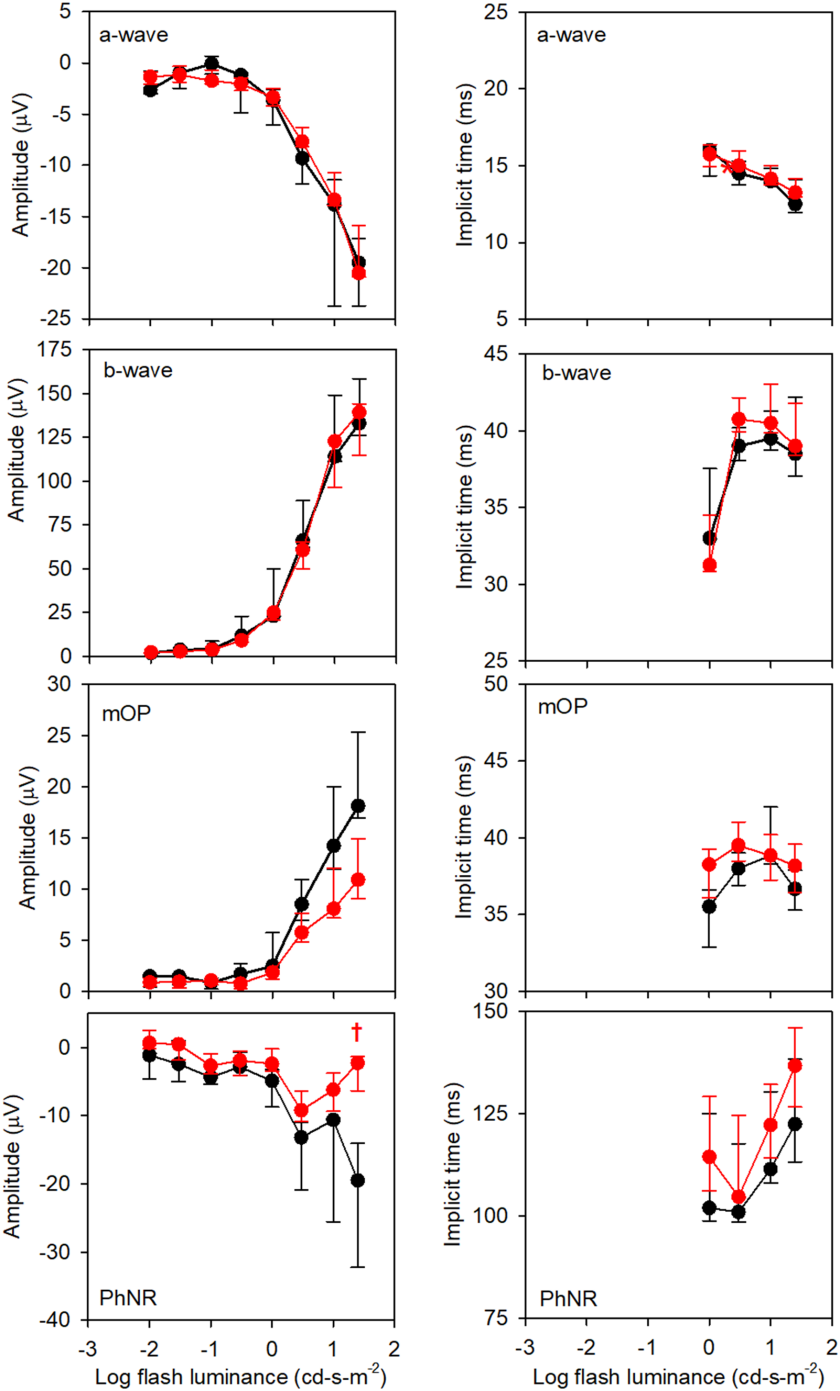
Median response amplitude (left column) and implicit time (right column) obtained under light-adapted conditions. All other conventions are as in Fig. 2.

Figure 5 shows the median amplitudes and ITs for the WT and 5xFAD mice under LA conditions. In contrast to the results obtained under DA conditions, the 5xFAD and WT ERGs were generally similar under LA conditions. In this figure, IT measurements for flash luminances below 1.0 cd-s-m^−2^ are not shown, as these measurements were not reliable given the small or absent response amplitude. Likewise, only flash luminances of 1.0 cd-s-m^−2^ and higher were included in the LQMMs. The first row of Fig. 5 shows that the median a-wave amplitudes and ITs for the WT and 5xFAD mice were highly similar, and the LQMM indicated no significant difference between the groups (median reduction of 1.66 µV, *p* = 0.28; median delay of 0.28 ms, *p* = 0.47). There was also no significance difference between the 5xFAD and WT mice for measurements made at a fixed time point (5 ms) following the flash (median difference of 0.33 µV; *p* = 0.50). The second row of Fig. 5 shows that the median b-wave amplitude for the 5xFAD mice was similar to that of the WT mice (median difference of 1.73 µV; *p* = 0.85) and there was no significant difference in b-wave IT (median difference of 0.66 ms; *p* = 0.46). The third row shows that the median OP amplitude was lower for the 5xFAD mice compared to the WT mice for the three highest flash lumainces, but these differences did not reach statistical significance (median difference of 3.39 µV; *p* = 0.08). There were also small delays in the median OP IT for the 5xFAD mice, but these delays also failed to achieve statistical significance (median difference of 1.53 ms, *p* = 0.08). The fourth row shows that the median PhNR amplitude was reduced and the response was delayed for the 5xFAD mice relative to the WT mice (median reduction of 6.79 µV, *p* = 0.009; median delay of 13.36 ms, *p* = 0.04). LQMs indicated that the WT and 5xFAD amplitude differed significantly only for the 25 cd-s-m^−2^ flash luminance (17.7 µV median reduction; *p* = 0.0006; indicated by the dagger). Preliminary analyses performed in a subset of animals (Supplementary Materials) showed a marginally non-significant nonlinear correlation between PhNR amplitude and Aβ42 concentration (Spearman’s ρ = -0.59, *p* = 0.051).

## Discussion

The purpose of the present study was to characterize the full-field ERG under both dark- and light-adapted conditions in 5xFAD mice to provide insight into rod- and cone-pathway dysfunction in these animals. The results show that (1) the ERG can be abnormal in 5xFAD mice, with the greatest deficits arising at post-receptor sites under dark-adapted conditions; (2) post-receptor abnormalities are observed under light-adapted conditions in the 5xFAD mice, but the abnormalities were generally less apparent relative to those observed under dark-adapted conditions and were restricted to ganglion cell dysfunction (inferred from measurement of the PhNR).

To our knowledge, this is the first report of full-field ERGs under light-adapted conditions in the 5xFAD mouse. The light-adapted a- and b-waves were normal in these animals, suggesting that cone photoreceptor function and signal transmission to second order neurons (ON and OFF bipolar cells) are not affected in young 5xFAD mice. This is consistent with Joly et al.^23^ who reported normal photopic a- and b-wave amplitudes in the APP_swe_/PS1ΔE9 mouse model of AD at 5 months of age. Interestingly, these mice had supernormal photopic responses at 13 months of age. In contrast to the normal a- and b-wave responses, we show a loss of PhNR amplitude for high flash luminance, suggesting retinal ganglion cell abnormalities. The relationship between PhNR amplitude and flash luminance was complex for both the 5xFAD and WT mice, consistent with previous data^29^. As shown in Fig. 5 (lower left panel), PhNR amplitude increased as flash luminance increased from 1 to 3 cd-s-m^−2^ for both the 5xFAD and WT mice. For the 5xFAD mice, the amplitude decreased at higher retinal illuminance, consistent with the “photopic hill” phenomenon that is discussed elsewhere (i.e. as stimulus luminance is increased, light-adapted PhNR and b-wave amplitude initially increase, then decrease for flashes in the higher photopic range)^38,39^. For the WT mice, the “photopic hill” was less apparent. The different pattern of PhNR data for the 5xFAD and WT mice may be related to altered ON/OFF pathway activity in the 5xFAD mice, as the photopic hill is thought to be related to the relative contributions of these pathways^38^. However, it should be noted that the PhNR measurements shown in Fig. 5 are generally small and variable among animals. Further work is needed to evaluate the relative effects of AD on the ON and OFF pathways.

The present report shows post-receptor abnormalities under dark-adapted conditions, consistent with Do et al.^21^ who also reported post-receptor abnormalities (b-wave amplitude loss) under dark-adapted conditions in a small sample of 6-month-old 5xFAD mice. Do et al.^21^ recorded responses elicited by low luminance stimuli (0.0001 to 0.075 cd-s-m^−2^), which does not readily allow for analysis of the a-wave. Criscuolo et al.^18^ measured dark-adapted full-field ERGs in a small sample of 5xFAD mice (1 to 6 months of age) at stimulus luminances of 0.1 and 20 cd-s-m^−2^. For both flash luminances, the average 5xFAD b-wave was smaller than that of the WT mice, but the difference between groups was not statistically significant. Criscuolo et al.^18^ also reported reduced pERG amplitude in the same mice, suggesting RGC dysfunction. Likewise, Lim et al.^22^ showed small b-wave amplitude losses in the 5xFAD mice at 6 and 12 months of age that did not meet the threshold for statistical significance. For older 5xFAD mice (17 months) they reported that the b-wave amplitude elicited by a high luminance flash was significantly reduced, and reductions in the a-wave were also noted^22^. Lim et al.^22^ found the scotopic threshold response to be reduced at all ages, which also suggested RGC dysfunction. Taken together, the results of the present report, as well as those of previous reports^18,21,22^, indicate post-receptor retinal abnormalities in the 5xFAD model of AD under dark-adapted conditions. Our results suggest that post-receptor function is selectively affected in the 5xFAD mouse and that the b-wave abnormalities are not secondary to rod photoreceptor dysfunction.

Although the dark-adapted a-wave amplitude was normal in the 5xFAD mouse, the timing of the a-wave trough was delayed; however, this was observed only for low flash luminances (Fig. 3). The explanation for the delay at low luminances is not entirely clear, but it may be related to a delayed onset of the bipolar cell response (b-wave onset delay). A second possibility is that the delay is also present at higher stimulus luminance, but it is masked by other contributions to the a-wave that occur at high flash luminances. Careful inspection of Fig. 3 shows that the a-wave delay disappears at the stimulus luminance that produces a shape change in the a-wave. That is, increasing the flash luminance from 1 to 3 cd-s-m^−2^ results in a squaring of the a-wave shape and the disappearance of the 5xFAD a-wave delay. For higher flash strengths (10 and 25 cd-s-m^−2^), the a-wave develops a broad trough and a “nose” within the trough becomes prominent that is thought to be generated by capacitive currents from the outer nuclear layer^40^. Additional work is needed to determine the source of the a-wave delay that is observed at low flash luminances in the 5xFAD mouse and why it may disappear for high luminance flashes.

There have been relatively few reports of retinal electrophysiology in human AD patients^3,5–10^, but the results are generally consistent with the limited data available in 5xFAD mice. Specifically, under light-adapted conditions, we show reduced PhNR amplitude, which is largely a measure of RGC function. Similarly, reduced PhNR amplitude in human AD subjects has also been reported recently^41^. Likewise, there are reports of pERG amplitude reduction and delays in human AD subjects^3,5–10^, which also indicate RGC abnormalities, consistent with previous 5xFAD mouse data^18^. Thus, electrophysiological measures of inner-retina function may be best suited for identifying biomarkers of AD in future clinical studies.

There are limitations of this study that should be considered in the interpretation of the data. First, given that the WT mice were not littermates of the 5xFAD mice, there could be genetic differences between these groups, other than the APP and PSEN1 mutations. This is particularly the case for the four WT mice obtained from Charles River Laboratories, which were not screened for rd1 and rd8 mutations. However, the ERGs from these four mice did not differ in any obvious way from the 9 WT mice obtained from The Jackson Laboratory. Second, we provide preliminary data relating ERG amplitude and Aβ42 concentration from a small sample; this must be repeated in a larger sample before definitive conclusions can be drawn. Finally, it is conceivable that the typical ketamine/xylazine anesthesia could have different effects on retinal function in the WT and 5xFAD mice (e.g. lower retinal blood oxygen saturation in the 5xFAD mouse). Although this is unlikely to be a major concern in our young mice, future studies using isoflurane anesthesia are needed to compare findings with the present dataset.

In summary, full-field ERG abnormalities are apparent in the 5xFAD mouse model of AD, with the greatest effect on post-receptor function under dark-adapted conditions. The results show that the post-receptor retinal dysfunction in young 5xFAD mice is not likely to be secondary to photoreceptor dysfunction. These results indicate that retinal electrophysiology may be a useful tool for evaluating neural dysfunction in AD. In future work, it will be of interest to determine the extent to which dysfunction of the neural retina, assessed by ERG, corresponds to cortical dysfunction and the associated cognitive impairments.

## Supplementary Information

Supplementary Information
