# Supplementary material for: Rod pathway and cone pathway retinal dysfunction in the 5xFAD mouse model of Alzheimer’s disease
Source: Sci Rep. 2021 Mar 1;11:4824. doi: 10.1038/s41598-021-84318-2 (PMC7921657; doi:10.1038/s41598-021-84318-2)
Supplement: Supplementary file 1 — Supplementary Information [file 41598_2021_84318_MOESM1_ESM.docx]

**Rod pathway and cone pathway retinal dysfunction**

**in the 5xFAD mouse model of Alzheimer’s disease**

J. Jason McAnany^1^, Nathanael Matei^2^, Yi-Fan Chen^3^, Karen Liu^1^,

Jason C. Park^1^, Mahnaz Shahidi^2*^

^1^ Department of Ophthalmology and Visual Sciences, University of Illinois at Chicago, Chicago, Illinois, USA

^2^ Department of Ophthalmology, University of Southern California, Los Angeles, California, USA

^3^ Center for Clinical and Translational Science, University of Illinois at Chicago, Chicago, Illinois, USA.

**Supplementary Material**

**(Methods and Figure)**

Aβ analysis

Amyloid protein was quantified using a commercially available enzyme-linked immunosorbent assay (ELISA) kit to determine the retinal concentration of Aβ42. The focus was on Aβ42 because 5xFAD mice almost exclusively generate Aβ42 that rapidly accumulates at high levels in the brain. The Aβ42 analysis used in the present study is described in detail elsewhere.[^1^](#_ENREF_1) In brief, three WT mice and eight 5xFAD mice were perfused with cold PBS solution (pH 7.4) by intracardiac injection. The retina was dissected, snap frozen in liquid nitrogen, and stored at -80°C. The retina was suspended in 70 µl of RIPA lysis buffer (Santa Cruz Biotechnology), sonicated, and centrifuged at 14,000xg at 4°C for 30 min. The supernatant was used for the ELISAs. The protein concentration was determined using a BCA protein assay kit (23225; ThermoFisher Scientific). Aβ42 levels in the extracts were quantified using an ELISA kit: Human Aβ42 ELISA kit (KHB3441; Invitrogen). A standard curve for Aβ42 was determined in all experiments using standards provided by the manufacturer. Duplicate samples were used for the Aβ42 experiments.

Figure S1 shows the results of the pilot study designed to evaluate the relationship between Aβ42 concentration and ERG amplitude. The top panel shows the dark-adapted b-wave amplitude for the 3.0 cd-s-m^2^ flash (a standard flash luminance) as a function of the normalized Aβ42 concentration (pg/mg) for the three WT (black) and eight 5xFAD (red) mice. The curve is an exponential decay function fit to the data. ERG b-wave amplitude tended to be larger for animals with lower concentrations of Aβ42. However, for Aβ42 concentrations greater than approximately 6 pg/mg, amplitude was generally independent of Aβ42 concentration. There was a significant nonlinear correlation between b-wave amplitude and Aβ42 concentration (Spearman’s ρ = -0.68, p = 0.019). Note that the correlation was not statistically significant when performed on the 5xFAD data alone (Spearman’s ρ = -0.17, p = 0.66). The bottom panel shows the light-adapted PhNR amplitude for the 25.0 cd-s-m^2^ flash (the highest flash luminance that elicited the largest PhNR) as a function of the normalized Aβ42 concentration (pg/mg). The PhNR amplitude tended to be larger for animals with lower concentrations of Aβ42, consistent with the b-wave findings. For Aβ42 concentrations greater than approximately 4 pg/mg, amplitude was generally independent of Aβ42 concentration. There was a marginally non-significant nonlinear correlation between PhNR amplitude and Aβ42 concentration (Spearman’s ρ = -0.59, p = 0.051). The correlation was not statistically significant when performed on the 5xFAD data alone (Spearman’s ρ = -0.07, p = 0.84).





Figure S1: ERG amplitude is plotted as a function of normalized Aβ42 concentration for three WT mice (black) and eight 5xFAD mice (red). ERG amplitude data are shown for the dark-adapted b-wave (3.0 cd-s-m^2^) in the top panel and for the light-adapted PhNR (25.0 cd-s-m^2^) in the bottom panel. The black line is an exponential function fit to the dataset.

1 Koronyo, Y. *et al.* Therapeutic effects of glatiramer acetate and grafted CD115(+) monocytes in a mouse model of Alzheimer's disease. *Brain : a journal of neurology* **138**, 2399-2422, doi:10.1093/brain/awv150 (2015).
